# Parameter Search Algorithms for Microwave Radar-Based Breast Imaging: Focal Quality Metrics as Fitness Functions

**DOI:** 10.3390/s17122823

**Published:** 2017-12-06

**Authors:** Declan O’Loughlin, Bárbara L. Oliveira, Muhammad Adnan Elahi, Martin Glavin, Edward Jones, Milica Popović, Martin O’Halloran

**Affiliations:** 1 Electrical and Electronic Engineering, National University of Ireland Galway, H91 TK33 Galway, Ireland; b.oliveira1@nuigalway.ie (B.L.O.); adnan.elahi@nuigalway.ie (M.A.E.); martin.glavin@nuigalway.ie (M.G.); edward.jones@nuigalway.ie (E.J.); martin.ohalloran@nuigalway.ie (M.O.); 2 McGill University, Montréal, QC, Canada H3A 0G4; milica.popovich@mcgill.ca

**Keywords:** biomedical electromagnetic imaging, microwave imaging, ultrawideband radar

## Abstract

Inaccurate estimation of average dielectric properties can have a tangible impact on microwave radar-based breast images. Despite this, recent patient imaging studies have used a fixed estimate although this is known to vary from patient to patient. Parameter search algorithms are a promising technique for estimating the average dielectric properties from the reconstructed microwave images themselves without additional hardware. In this work, qualities of accurately reconstructed images are identified from point spread functions. As the qualities of accurately reconstructed microwave images are similar to the qualities of focused microscopic and photographic images, this work proposes the use of focal quality metrics for average dielectric property estimation. The robustness of the parameter search is evaluated using experimental dielectrically heterogeneous phantoms on the three-dimensional volumetric image. Based on a very broad initial estimate of the average dielectric properties, this paper shows how these metrics can be used as suitable fitness functions in parameter search algorithms to reconstruct clear and focused microwave radar images.

## 1. Introduction

In recent years, microwave imaging has shown promising results in early breast imaging clinical trials. In particular, an ongoing study with over 200 patients shows sensitivities equivalent to mammography, and slightly higher than mammography in dense breasts [1,2]. Other studies have analysed the variability of measurements of healthy volunteers over time-frames of two to eight months and analysed the comfort levels of patients [3]. Previous studies have also shown for eight patients with and without disease that the reconstructed images are consistent with the clinical history of the patient [4].

In general, microwave radar imaging for breast cancer can be considered analogous to synthetic aperture radar, where a synthetic aperture array of non-directional antennas sequentially illuminates the imaging domain and backscattered signals are collected either at the transmitting antenna (monostatic) or at the transmitting antenna and other receivers (multistatic). These backscattered signals are then synthetically focused to points within the imaging domain and the energy of the summed signal used as the intensity of the point. At points where dielectric scatterers are located, coherent addition occurs resulting in a larger energy than the surrounding area.

This technique relies on a number of assumptions:

1. that sufficient contrast exists between cancerous and healthy tissues [5];
2. that large reflections due to the skin can be isolated and removed [6,7,8,9];
3. and that the backscattered signals can accurately be synthetically focused to points within the imaging domain [10,11,12,13,14].

No consensus exists in the literature on the expected contrast in dielectric properties between cancerous and healthy breast tissues in the microwave frequency band from 0.5 to 10GHz. Initial studies indicated an expected contrast in dielectric properties of cancerous and healthy tissues of between 2.3:1 and 10:1, and extensive reviews of these early studies have been published [15,16,17,18,19]. However, in 2007, the largest and most comprehensive study to date suggested the contrast in dielectric properties of cancerous and healthy breast tissues could be as low as 1.1:1 [20,21]. This motivated research of suitable contrast agents which could be used for differential imaging and a number of promising results have been demonstrated in the literature [22,23,24,25,26,27]. Additionally, a number of studies on dielectric properties measurement have examined factors that could impact the measurement, such as differences between *ex vivo* and *in vivo* dielectric properties measurement [28,29,30,31]; histological analysis of the tissue samples [32]; and the sensing depth of the metrological techniques used [33,34]. These recent studies on dielectric properties measurement, in addition to promising reported results from ongoing patient imaging trials [1,2], indicate that the expected contrast in the context of microwave imaging, might not be as low as measured in [20,21].

This work considers the assumption that backscattered signals can accurately be synthetically focused to points within the imaging domain. Specifically, this work analyses the effect that incorrectly estimating the average dielectric properties has on image quality. The average dielectric properties estimate is the main contributing factor to focusing accuracy [35].

To date, one effective average dielectric property has been chosen *a priori* for patient imaging studies [1,2,3,4]. However, this parameter is known to be patient-specific [13,36]. Due to the importance of the effective average dielectric properties as an imaging parameter, a number of methods have been considered to estimate the effective average dielectric properties accurately in simulation and experimental studies [10,14,36,37]. There are two primary means of estimating the effective average dielectric properties:

- estimating from signals that have propagated through the imaging domain;
- and via parameter search based on properties of the reconstructed images.

Estimation from time-of-flight assumes that the properties of the chosen paths are representative of the volume as a whole. In contrast, parameter search algorithms assume that the properties of images with incorrectly estimated effective average dielectric properties are different to those of correctly focused images. Algorithms based on parameter search are useful because they are largely independent of the imaging hardware and configuration, and are not susceptible to multipath propagation within the imaging domain.

Two main metrics for parameter search have been proposed for microwave imaging for both stroke and breast cancer detection based on

- maximising the response in a 4 cm^2^ area while minimising the energy outside this region [38,39];
- or rewarding images with one strong response and penalising other large or strong responses [14,40].

Based on the properties of incorrectly estimated images observed from point spread functions (PSFs), focal quality metrics (FQMs) are proposed as suitable and effective fitness functions that can be used in parameter search algorithms for estimating the average dielectric properties. Twenty-three commonly used FQMs are investigated. FQMs are commonly used in microscopy and digital photography for focal length optimisation [41].

FQMs have been identified as potential fitness functions for parameter search algorithms in simplified, numerical simulations using ideal artefact removal algorithms [42,43,44]. This work analyses FQMs in more realistic experimental test cases, using a practical artefact removal algorithm and diverse heterogeneous phantom set with between 10 and 40% fibroglandular structures by volume. This allows the parameter search algorithm to be tested in the presence of experimental noise and hardware artefacts. Additionally, a parameter search algorithm is applied to the full three-dimensional images, unlike the two-dimensional images analysed in [44].

The rest of this paper is structured as follows: Section 2 describes the parameter search algorithm in detail and how the properties of high quality images are determined; Section 3 describes common FQMs used in focusing and analysed in detail in this work and Section 4 describes the experimental setup used to validate the algorithms. Section 5 shows the results of the theoretical analysis and experimental validation; and Section 6 concludes the paper, identifying suitable FQMs for the estimation of average dielectric properties.

## 2. Methods

A microwave imaging prototype can be thought of as a synthetic aperture radar system where the imaging volume is illuminated sequentially from antennas placed on a surface, A, and backscattered reflections are recorded from antennas placed on a surface, $A^{\prime }$. In the monostatic case, where reflections are recorded at the same transmitting locations in the frequency range, Ω, the Delay-and-Sum (DAS) [4,6,7,45,46,47,48,49,50,51,52,53,54,55,56,57,58] beamformer can we written as (in the frequency domain):

(1) $$I\left(r\right)=\int_{\Omega }\int_{A}S_{s,s}^{\prime }\left(\omega \right)\exp j\omega \tau_{s}\left(r,\;\omega \right)ds\;d\omega$$

where $\tau_{s}\left(r,\;\omega \right)=\int_{C(s,r)}\frac{1}{c(r,\omega )}ds$ is the propagation delay from the transmitting antenna to the point of interest, r, and back to the receiving antenna along the propagation path, C(s,r), for the frequency and spatial-dependent propagation speed, c(r,ω). $S_{s,s}$ is the backscattered signals for the antenna at s.

Many uncertainties affect propagation delay estimation:

- the exact propagation path, C(s,r), is not known as the tissue composition of the imaging volume is unknown in a screening context;
- human breast tissues are dispersive, but the frequency-dependent propagation speed for each tissue is not known exactly, c(ω);
- the propagation speed, c(r,ω), along the propagation path is also unknown as this depends on the exact tissue composition of the imaging volume.

In practice, certain simplifying assumptions are made in the imaging operator:

- the propagation path is assumed as the straight-line path from the antenna, s, to the point of interest, r. This has been found to have a minimal impact on accuracy, at worst 3 mm [35];
- the propagation speed is generally assumed to be defined at the centre frequency of the illumination pulse, $\omega_{c}$;
- the propagation speed is assumed to not vary spatially in the entire imaging domain. Although a preliminary numerical study indicated that localisation accuracy could be improved by adapting the propagation delay depending on paths within the breast, this is not practical in realistic scenarios [11].

The above simplifying assumptions have been used in all microwave patient studies to date [1,3,4], and this work further investigates if the propagation speed can be adapted per patient using parameter search algorithms.

As biological tissues are non-magnetic, the effective propagation speed (at the centre frequency of the illumination pulse) can be written as $c\left(r\right)=\frac{c_{0}}{\sqrt{\varepsilon_{r}^{\prime }}}$ where $c_{0}$ is the speed of light and $\varepsilon_{r}^{\prime }$ is the effective average relative permittivity of the medium at the centre frequency of illumination. The effective average relative permittivity is a weighted average of the average relative permittivity of the entire breast. Thus, the propagation speed can be approximated as $\tau_{s}\left(r,\varepsilon_{r}\right)=2\frac{\sqrt{\varepsilon_{r}^{\prime }}∥r-s∥}{c_{0}}$ Therefore, Equation (1) can be represented as follows:

(2) $$I_{\varepsilon_{r}^{\prime }}\left(r\right)=\int_{\Omega }\int_{A}S_{s,s}\left(\omega \right)\exp j\omega \tau_{s}\left(r,\;\varepsilon_{r}^{\prime }\right)ds\;d\omega$$

**Figure 1:**
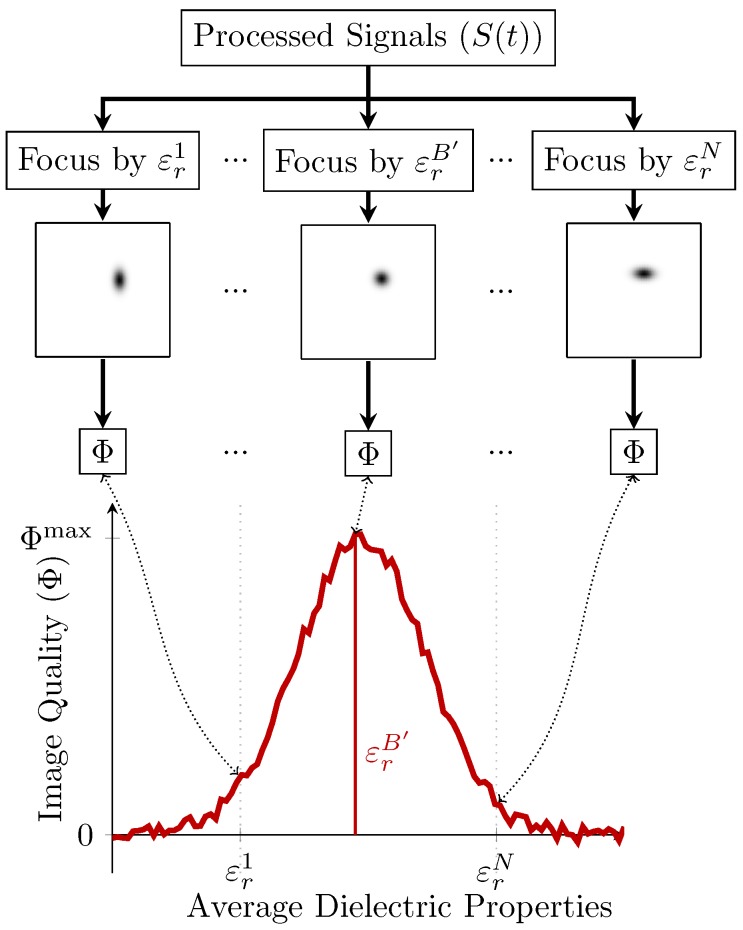
Block diagram of the proposed imaging system. Focal quality metrics are used in a parameter-search algorithm to identify the best-case average dielectric properties, $\varepsilon_{r}^{B^{\prime }}$. The proposed algorithm is described: from a set of images reconstructed with different average dielectric properties estimates, select the image that the measure of image quality, Φ, weights most highly.

This paper investigates suitable fitness functions to use in parameter search algorithms to estimate the effective average dielectric properties, $\varepsilon_{r}^{\prime }$, summarised in Figure 1:

1. A set of backscattered signals, S(t), is beamformed into the set of images, $I=\{I_{\varepsilon_{r}}\;|\;\varepsilon_{r}\in \varepsilon_{r}^{\mathrm{range}}\}$ using a range of assumed average dielectric properties $\varepsilon_{r}^{\mathrm{range}}$;
2. Given a measure of image quality, Φ, apply the measure to the the set of images to determine the relative quality of the images where $\Phi \left(I_{\varepsilon_{r}}\right)=\left\{\Phi \left(I_{\varepsilon_{r}}\right)\;|\;I_{\varepsilon_{r}}\;\in \;I\right\}$;
3. Determine the optimal average dielectric properties, and hence the optimal image, by optimising the relative quality curve, $\Phi (I_{\varepsilon_{r}})$, such that the estimated best-case average dielectric properties, $\varepsilon_{r}^{B^{\prime }}$, are determined as, $\varepsilon_{r}^{B^{\prime }}=\;\arg\;\max\;\Phi \left(\varepsilon_{r}\right)$.

### 2.1. Effect of Incorrect Estimation

The effect of an incorrect estimation of the effective average dielectric properties is considered by analysing the PSF. Firstly, a simplified skinless two-dimensional environment is considered, which is then compared to the imaging system described in this paper. The simplified environment is described in [59], where the backscattered signals are described as:

(3) $$S_{s,s}^{\prime }\left(\omega \right)=\frac{j\omega \sqrt{\varepsilon_{r}}}{2\pi c_{0}}P\left(\omega \right)\int_{D}\frac{1}{∥r-s∥}\exp\frac{-2j\omega \sqrt{\varepsilon_{r}}}{∥r-s∥}\chi \left(r\right)dr$$

Given a point source such that $\chi \left(r\right)=\chi \delta \left(r-r^{T}\right)$ where $r^{T}$ is the scatterer location, assuming without loss of generality that $\left|P(\omega )\right|=1\;\forall \;\omega \in \Omega$ and substituting Equation (3) into Equation (2) results in the following expression for the point spread function of the simplified system:

(4) $$I_{\varepsilon_{r}^{\prime }}\;\left(r\right)=\;\alpha \;\;\;\int_{\Omega }\;\;\omega \;\;\;\int_{A}\;\;\frac{\exp\;\frac{2j\omega }{c_{0}}\;\left[\sqrt{\varepsilon_{r}^{\prime }}∥r\;-\;s∥\;-\;\sqrt{\varepsilon_{r}}∥r^{T}\;\;-\;s∥\right]}{∥r-s∥}dsd\omega$$

where $\alpha =\frac{\chi j\sqrt{\varepsilon_{r}}}{2\pi c_{0}}$ is a constant proportional to the contrast of the dielectric scatterer and inversely proportional to the propagation speed in the medium.

Assuming initially that $\sqrt{\varepsilon_{r}}=\sqrt{\varepsilon_{r}^{\prime }}$, Equation (4) is maximised when the distances to the points are equal to the distances to the scatterer for each antenna (i.e., with the effective average dielectric properties estimate, the image maximum is at the scatterer location). However, as the ratio between the effective average dielectric properties and the true average dielectric properties, $\sqrt{\frac{\varepsilon_{r}^{\prime }}{\varepsilon_{r}}}$, changes, the distance for a given antenna to maximise the exponential changes proportionally. Due to radial spreading (and additionally due to losses in a realistic scenario), this means that the maximum intensity of the image moves towards the antenna closest to the scatterer. This can be observed in Section 5 by examining the one-dimensional PSFs generated by integrating Equation (4) numerically.

The point spread function of the system described in Section 4 is also measured experimentally. A dielectric point source can be approximated by an object that has a maximum diameter less than half the wavelength in the object, $d_{\max}<\frac{1}{2}\lambda_{\min}$ [35]. For a spherical scatterer with relative permittivity, $60\leq \varepsilon_{r}^{\mathrm{target}}\leq 74$ such as tumours, the maximum diameter should be less than, $d_{\max}<5\;\mathrm{mm}$, if the maximum frequency in the reconstruction is, $f_{\max}=3\;\mathrm{GHz}$. The experimental point spread functions are compared with the simplified theoretical analysis and the suitability of the metrics is assessed on the point spread functions.

### 2.2. Evaluating Suitable Metrics

Four evaluation criteria are considered in this work to assess the suitability of the various metrics tested.

1. the accuracy ($\Delta \varepsilon_{r}$): the difference between the best-case effective average dielectric properties, $\varepsilon_{r}^{B^{\prime }}$, and the true average dielectric properties, $\varepsilon_{r}^{B}$;
2. the localisation error, Δr: the difference between the apparent location of the scatterer when reconstructing images with the effective average dielectric properties and the location when reconstructing images with the true average dielectric properties;
3. the signal-to-clutter ratio (SCR) of the reconstructed image, $\mathrm{SCR}(I_{\varepsilon_{r}^{B^{\prime }}})$;
4. the signal-to-mean ratio (SMR) of the reconstructed image, $\mathrm{SMR}(I_{\varepsilon_{r}^{B^{\prime }}})$.

Smaller values of $\Delta \varepsilon_{r}$ and Δr are better, while larger values of $\mathrm{SCR}(I_{\varepsilon_{r}^{B^{\prime }}})$ and $\mathrm{SMR}(I_{\varepsilon_{r}^{B^{\prime }}})$ are better.

## 3. Focal Quality Metrics

This section describes the FQMs evaluated for this study. FQMs have been used in multiple areas to optimise image quality, for example, microscopy [60,61,62]; telescopy [63,64]; digital still cameras [65,66,67]; and digital video [68]. In this paper, FQMs are evaluated in detail as suitable fitness functions for parameter search algorithms for microwave radar breast imaging.

In general, FQMs estimate the high frequency content of the image, as clear and focused images tend to feature more high-frequency content [69,70]. In the context of microwave radar breast imaging, this means energy is concentrated at scatterer locations and not distributed around the image in clutter. FQMs can be broadly classified based on their method of action, that is, how they estimate the high frequency content of the image.

All FQMs used in this study are summarised in Table 1 divided into five FQM families based on the following:

- the Discrete Cosine Transform ($\phi^{F}$);
- image gradient ($\phi^{G}$);
- Laplacian approximation ($\phi^{L}$);
- image statistics ($\phi^{S}$);
- and the Discrete Wavelet Transform ($\phi^{W}$).

The discrete cosine transform (DCT) is a Fourier transform that uses cosines as basis functions. The DCT directly measures the frequency content of the image, as an estimation of the image quality. The energy of the AC components of the DCT (which is an estimate of the variance of the luminance of an image) has been used as a focal quality measure [68]. However, it was found that the energy of the AC components is sensitive to image contrast and that the ratio of the AC energy to the DC energy is more homologous [71]. Different window sizes have also been used, either eight pixels square [41,68,72] or four pixels square [71].

Gradient-based FQMs, $\phi^{G}$, use approximations of the gradient or the first-derivative of the image to estimate the high-frequency content and hence the image quality. Differentiation is considered analogous to high-pass filtering, so these methods reward high-frequency content in the image. Different approximations of the gradient have been used: first-order differences, $I_{X}^{D}$, [66,73,74,77]; Brenner gradient, $I_{X}^{B}$, [60,73,74,75]; Gaussian derivative, $I_{X}^{G}$; and Tenengrad, $I_{X}^{T}$, [69,73,74,77,80,81,90].

Different combinations of the components of the gradient have also been looked at: one-dimensional approximations [60,74,75,79]; maximum component approximations [41,73]; and component sum [66,69,72,74,77,78,80,81,90].

Additionally, prior to summation, either the absolute value of the components of the gradient [73,74] or the squared value of the components of the gradient, [60,66,69,72,73,74,75,77,78,79,80,81,90] can be used.

Laplacian-based FQMs, $\phi^{L}$, use second-order differentiation to reward higher frequency content in the image and hence reward images of higher quality. The second-order derivative is approximated by convolving a Laplacian kernel (two-dimensional) with the image, where the kernel, *L*, is given by:

(5) $$L=\frac{1}{6}\left(\begin{array}{ccc} 1 & 4 & 1\\ 4 & -20 & 4\\ 1 & 4 & 1 \end{array}\right)$$

The Laplacian kernel can also be applied in each direction independently, where the kernels in the *x* and *y* directions are given by

(6) $$L_{x}=L_{y}^{T}=\left(\begin{array}{ccc} -1 & 2 & -1 \end{array}\right)$$

Finally, the Laplacian kernel can also be estimated along the diagonals where the kernels along the two diagonals, $L_{d}$ and $L_{d}^{\prime }$, are given by

(7) $$L_{d}\;=\;\frac{1}{\sqrt{2}}\left(\begin{array}{ccc} 0 & 0 & 1\\ 0 & -2 & 0\\ 1 & 0 & 0 \end{array}\right)\;\;,\;L_{d}^{\prime }\;=\;\frac{1}{\sqrt{2}}\left(\begin{array}{ccc} 1 & 0 & 0\\ 0 & -2 & 0\\ 0 & 0 & 1 \end{array}\right)$$

Statistics-based metrics, $\phi^{S}$, analyse the distribution of values of the image or the histogram of the image, such as

- variance;
- contrast;
- entropy;
- and the central moment.

Wavelet-based metrics, $\phi^{W}$, use the discrete wavelet transform (DWT) to describe the frequency content of the image and reward images of higher quality. The DWT decomposes the image into three detail sub-bands—$W_{\mathrm{LH}}^{1}$, $W_{\mathrm{HL}}^{1}$ and $W_{\mathrm{HH}}^{1}$—and the coarse approximation sub-band, $W_{\mathrm{LL}}^{1}$. To create higher-level transforms, the coarse approximation sub-band is successively decomposed.

## 4. Experimental Evaluation

This section describes the experimental measurement system and acquisition hardware. Additionally, the four breast phantoms and five tumour models used are presented.

A flexible microstrip antenna was used to collect backscattered data. A 16-element antenna array has previously been used in pilot clinical trials [3,91]. In this work, experimental data were collected using a 24-element hemispherical conformal antenna array, meaning that 276 independent, multistatic channels were available for imaging. This is an increase from the 120 independent multistatic channels used previously [3]. 24 antennas were chosen based on the available space in the radome, as the antenna footprint is approximately 4–5 cm${}^{2}$. The antennas were designed to be in contact with skin of $\varepsilon_{r}=30$. In this system, the antennas were placed in direct contact with the skin layer of the breast phantoms.

**Figure 2:**
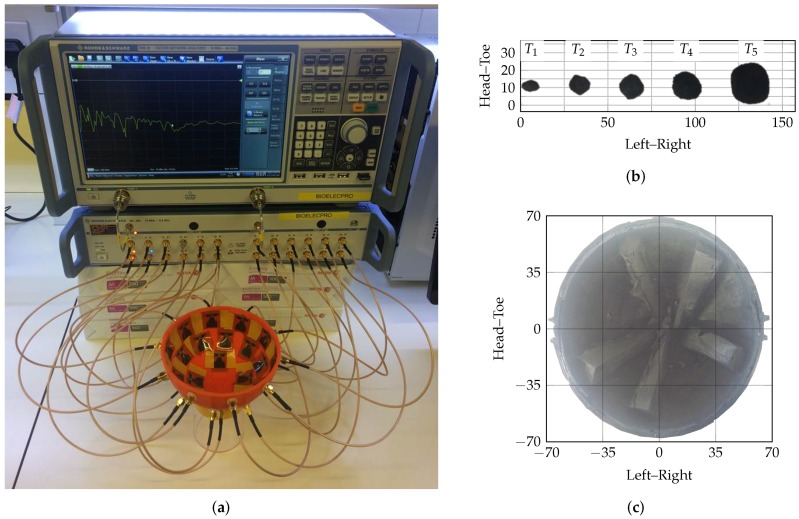
The acquisition system, example phantom and targets are shown here. (**a**) shows the 2-port VNA connected to the 24-port switching matrix. The antennas are shown housed in the 3D printed radome; (**b**) shows the five spherical and smooth tumour models used for evaluation of the FQMs; (**c**) shows the interior of the phantom with 10% glandular content. Three other similar phantoms with 0%, 20% and 30% glandular content were used in this study. All dimensions are in mm.

Fused deposition modelling (FDM) was used to fabricate a hemispherical radome to house the antennas. The radome, printed using polylactic acid (PLA) with an Ultimater 2+ Extended (Ultimaker, Geldermasen, The Netherlands), was designed with 24 holes to house the antennas. The antenna array is equally spaced across the hemisphere, as shown in Figure 2a.

Modular polyurethane breast phantoms and tumour models were fabricated according to [92,93,94]. A 2 mm skin layer was modelled with relative permittivity of $\varepsilon_{r}=30$, an interior fatty background was modelled with relative permittivity of $\varepsilon_{r}=6$, conical internal glandular structures were modelled with relative permittivity of between $40\leq \varepsilon_{r}\leq 50$ and tumours of $60\leq \varepsilon_{r}\leq 74$. All relative permittivity values are quoted at 3 GHz. Modelled tissue dielectric properties were chosen in accordance with [20,21], with the target tumour values representing the maximum values reported.

The tumour targets are shown in Figure 2b. Tumours of different levels of spiculation were fabricated and used to evaluate algorithm performance. The smallest spherical tumour (maximum diameter of 5 mm; labelled $T_{1}$ in Figure 2b) was used to evaluate the point spread function of this experimental system.

A ZNB40 2-port VNA and ZN-Z84 24-port switching matrix (Rohde and Schwartz GmbH, Munich, Germany) were used to collect all multistatic signals at 201 linearly spaced frequency points between 0.5 GHz and 8.5 GHz. The response for each channel was shaped by a Gaussian pulse modulated with a sine wave in the frequency-domain with centre frequency of 3 GHz and a bandwidth of 3 GHz. The shaped response was then transformed to the time-domain using the inverse Chirp-Z Transform and sampled at 80 GHz.

Rotational subtraction was used for artefact removal [95], to isolate the tumour response from the signal and reduce unwanted reflections. This technique has been successfully used in the largest microwave imaging clinical trial to date [1,2]. The antenna array was designed so that the rotated scan can be collected without any mechanical movement, reducing the overall patient scanning time to about 30 s. For example, antennas $s_{1}$, $s_{2}$, $s_{3}$ are in a concentric ring offset from each other by 36°, so the response for channel $S_{s_{1},s_{2}}$ is given by

(8) $$S_{s_{1},s_{2}}=S_{s_{1},s_{2}}^{\prime }-S_{s_{2},s_{3}}^{\prime }$$

where $S_{s,s^{\prime }}^{\prime }$ are the unprocessed backscattered reflections recorded at $s^{\prime }$ after transmitting on s.

Images were generated using the Delay-and-Sum beamformer [4,6,7,47,48,49,50,51,52,53,54,55,56,57]:

(9) $$I_{\varepsilon_{r}^{\prime }}\left(r\right)=\sum_{0}^{T}{\left(\sum_{c}^{N_{c}}S_{c}\left(t-\tau_{c}\left(r,\varepsilon_{r}^{\prime }\right)\right)\right)}^{2}$$

$S_{c}$ is the time-domain response as described above, $N_{c}$ is the number of multistatic channels and *T*, the window-length, is 330 ps, the length of the excitation pulse in the time-domain.

$\tau_{c}\left(r,\varepsilon_{r}^{\prime }\right)$, the propagation delay for each channel, is estimated based on the effective average dielectric properties $\varepsilon_{r}^{\prime }$, which is the parameter estimated in the parameter search algorithm. Forty-nine images, ranging from $\varepsilon_{r}=1$ to $\varepsilon_{r}=25$ were reconstructed (I) and the best-case image was selected using the metrics described in Section 2.

## 5. Results

The results are presented in multiple stages:

1. the effect of incorrectly estimating the effective average dielectric properties is analysed using simplified theoretical PSFs and then experimental PSFs;
2. next, promising FQMs from each family are selected by evaluating all FQMs described using a variety of targets in a homogeneous breast phantom;
3. finally, the best performing metrics in the homogeneous phantoms are analysed in increasingly complex and dielectrically heterogeneous scenarios using an experimental prototype imaging system.

### 5.1. Effect of Incorrect Parameter Estimation

**Figure 3:**
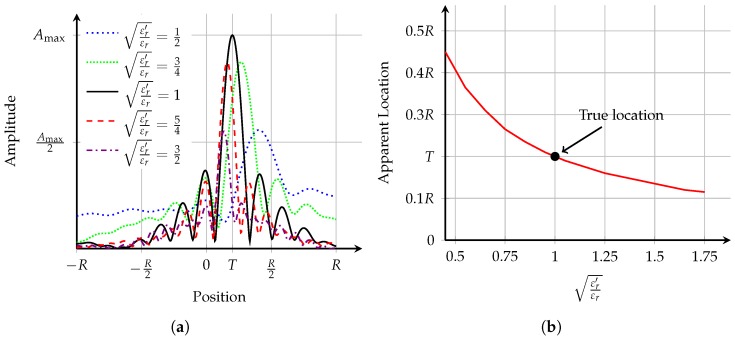
The theoretical point spread function (PSF) is analysed here. (**a**) shows the one-dimensional PSF for various values of $\sqrt{\frac{\varepsilon_{r}^{\prime }}{\varepsilon_{r}}}$. Localisation error increases as the difference between $\varepsilon_{r}^{\prime }$ and $\varepsilon_{r}$ grows. The number of sidelobes decreases as $\sqrt{\frac{\varepsilon_{r}^{\prime }}{\varepsilon_{r}}}$ decreases; (**b**) shows the apparent location of the scatterer as $\sqrt{\frac{\varepsilon_{r}^{\prime }}{\varepsilon_{r}}}$ varies; As the effective average dielectric properties are overestimated ($\sqrt{\frac{\varepsilon_{r}^{\prime }}{\varepsilon_{r}}}>1$), the apparent location is closer to the centre (0) compared to the true location. As the effective average dielectric properties are underestimated ($\sqrt{\frac{\varepsilon_{r}^{\prime }}{\varepsilon_{r}}}<1$), the apparent location is closer to the skin (*R*) compared to the true location. In both (**a**,**b**), the true location is at T=0.2R.

The effects of incorrect average dielectric property estimation are first analysed using the theoretical PSFs in Figure 3. Figure 3a shows the PSF of the ideal system obtained by numerically integrating Equation (4). Additionally, the location of the strongest response in the image is shown in Figure 3b. The true location of the dielectric point scatterer is at T=0.2R.

A number of observations can be made from Figure 3:

- in general, the maximum amplitude of the PSF is when the effective average dielectric properties, $\varepsilon_{r}^{\prime }$, is equal to the true average dielectric properties, $\varepsilon_{r}$;
- if the effective average dielectric properties are underestimated (i.e., $\sqrt{\frac{\varepsilon_{r}^{\prime }}{\varepsilon_{r}}}<1$), the apparent location of the scatterer moves towards the edge of the imaging domain (closer to *R*). This localisation error is due to reflections appearing to come from closer than their true origin and the channels closest to the scatterer are dominant in the coherent summation;
- conversely, if the effective average dielectric properties are overestimated, the apparent location of the scatterer moves towards the centre of the imaging domain (closer to 0). This localisation error is due to reflections appearing to come from further away than their true origin;
- the number of sidelobes increases as the estimate of the effective average dielectric properties increases; in other words, there is higher spatial frequency content in PSFs with over-estimated effective average dielectric properties, $\sqrt{\frac{\varepsilon_{r}^{\prime }}{\varepsilon_{r}}}>1$;
- it can be seen that as the effective average dielectric properties are overestimated, the width of the peak decreases.
- the localisation error is greater when underestimating the effective average dielectric properties compared to overestimation.

**Figure 4:**
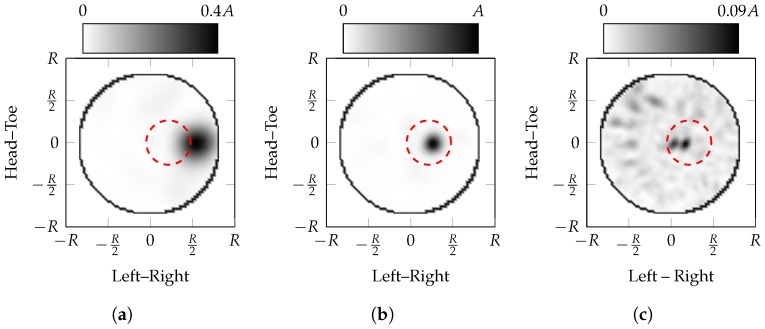
Coronal slices of the experimental PSF at the tumour location. The maximum intensity of images (**a**,**c**) are 40% and 9% of image (**b**). The normalised images with linear colour scales are displayed so that features can be more clearly identified. Images (**a–c**) are reconstructed with $\sqrt{\frac{\varepsilon_{r}^{\prime }}{\varepsilon_{r}}}\in \left\{0.5,1,1.5\right\}$ respectively. The location of the point scatterer is marked with the circle.

Coronal slices of the experimental PSF at the dielectric point scatterer location are shown in Figure 4. Figure 4a–c are reconstructed with effective average dielectric properties of $\varepsilon_{r}^{\prime }\in \left\{1.5,6,13.5\right\}$ respectively, where fatty breast interior has dielectric properties of $\varepsilon_{r}=6$. Hence, Figure 4a–c are reconstructed with $\sqrt{\frac{\varepsilon_{r}^{\prime }}{\varepsilon_{r}}}\in \left\{0.5,1,1.5\right\}$ respectively. Comparable trends can be observed in the PSF of the theoretical and experimental systems:

- the maximum amplitude of the images with incorrectly estimated effective average dielectric properties is much lower than the ideal image, 40% when underestimated and 9% when overestimated;
- the apparent location of the scatterer moves towards the edge of the imaging domain when the effective average dielectric properties are underestimated, i.e., $\sqrt{\frac{\varepsilon_{r}^{\prime }}{\varepsilon_{r}}}<1$;
- the apparent location of the scatterer moves towards the centre of the imaging domain when the effective average dielectric properties are overestimated, i.e., $\sqrt{\frac{\varepsilon_{r}^{\prime }}{\varepsilon_{r}}}>1$;
- Figure 4c has more clutter with greater magnitude than Figure 4a. This is similar to the theoretical case where the image reconstructed with overestimated effective average dielectric properties (i.e., $\sqrt{\frac{\varepsilon_{r}^{\prime }}{\varepsilon_{r}}}>1$) has higher spatial frequency content.
- the area of the response decreases as the estimated effective average dielectric properties increase.
- The localisation error when overestimating the properties is less than when underestimating the properties.

### 5.2. Initial Evaluation

To determine the most suitable FQMs, all FQMs were evaluated for the 5 targets in Figure 2b using the dielectrically homogeneous phantom. The mean value of the evaluation criteria ($\Delta \varepsilon_{r}$, Δr, $\mathrm{SMR}(I_{\varepsilon_{r}^{B^{\prime }}})$ and $\mathrm{SCR}(I_{\varepsilon_{r}^{B^{\prime }}})$) is shown in Table 2. Metrics are listed in order of rank for each method of action, and this rank is shown. Also shown is a global rank which is useful for comparing the different methods of action.

Of all the metrics analysed, two metrics perform very well as fitness functions: the Central Moment, $\phi_{\mathrm{ACM}}^{S}$, and the Gaussian Energy, $\phi_{\mathrm{GSS}}^{G}$. The Central Moment, $\phi_{\mathrm{ACM}}^{S}$, rewards images that are closest to the average dielectric properties, being on average within $\Delta \varepsilon_{r}=0.7$ of the known value of $\varepsilon_{r}=6$. However, the Gaussian Energy, $\phi_{\mathrm{GSS}}^{G}$, rewards images that are of a high quality, rewarding images that have the best localisation error, Δr, and the best clutter suppression, $\mathrm{SMR}(I_{\varepsilon_{r}^{B^{\prime }}})$ and $\mathrm{SCR}(I_{\varepsilon_{r}^{B^{\prime }}})$. Other metrics based on the gradient, $\phi^{G}$, or statistics, $\phi^{S}$, of the image also reward images of high quality; ten of the top eleven metrics use these methods of action.

All metrics based on the Laplacian of the image, $\phi^{L}$, perform very similarly, selecting images with almost the same accuracy, $\Delta \varepsilon_{r}$, localisation error, Δr, and clutter suppression, $\mathrm{SMR}(I_{\varepsilon_{r}^{B^{\prime }}})$ and $\mathrm{SCR}(I_{\varepsilon_{r}^{B^{\prime }}})$. Five more metrics perform very similarly to metrics based on the Laplacian, $\phi^{L}$: three based on the gradient of the image, $\phi^{G}$, the Tenengrad mean, $\phi_{M}^{T}$, the Squared Gradient, $\phi_{\mathrm{DMS}}^{G}$, and the Gradient Energy, $\phi_{\mathrm{GSS}}^{G}$; and two based on wavelet decomposition of the image, $\phi^{W}$, the Detail Variance, $\phi_{V}^{W}$, and the Absolute Detail Sum, $\phi_{\mathrm{AS}}^{W}$.

The two metrics based on the Fourier transform, $\phi^{F}$, do not perform well as fitness functions in these scenarios, identifying images with poor clutter suppression, $\mathrm{SMR}(I_{\varepsilon_{r}^{B^{\prime }}})$ and $\mathrm{SCR}(I_{\varepsilon_{r}^{B^{\prime }}})$. Additionally, the Fourier-based metrics, $\phi^{F}$, select images with localisation errors that are, on average, greater than 10 mm, Δr>10mm. As shown in Figure 4, images generated with underestimated effective average dielectric properties, $\sqrt{\frac{\varepsilon_{r}^{\prime }}{\varepsilon_{r}}}<1$, are characterised by large responses much closer to the skin than the true scatterer location. Metrics based on the Fourier transform, $\phi^{F}$, reward these images resulting in poor performance [44]. Metrics based on the Fourier transform, $\phi^{F}$, were first proposed for low-contrast images where they can be more effective [71], whereas the contrast for microwave radar images is higher. Although the AC–DC Reduced Ratio, $\phi_{\mathrm{RR}}^{F}$, performed better in noisy images than the AC–DC Ratio, $\phi_{R}^{F}$, in experimental images, that was not found for microwave radar images.

The Detail–Coarse Ratio, $\phi_{R}^{W}$, fails to reward any correct image. Similarly to metrics based on the Fourier transform, $\phi^{F}$, the Detail–Coarse Ratio, $\phi_{R}^{W}$ heavily rewards images generated with underestimated effective average dielectric properties, $\sqrt{\frac{\varepsilon_{r}^{\prime }}{\varepsilon_{r}}}<1$, such that it always selects images generated with effective average dielectric properties of free-space, $\varepsilon_{r}^{\prime }=1$.

Three suitable fitness functions were selected for further analysis in the subsequent sections: the Gaussian Energy, $\phi_{\mathrm{GSS}}^{G}$; the Modified Laplacian, $\phi_{M}^{L}$; the Central Moment, $\phi_{\mathrm{ACM}}^{S}$. The three FQMs metrics use three different methods of action based on the image gradient, the image Laplacian and statistic of the image respectively.

### 5.3. Detailed Analysis

Table 3 analyses the metrics selected in the previous section on five spherical targets of increasing diameter, d∈[5.3,20.2]mm, in phantoms with increasing volumes of heterogeneous tissues (10%, 20% and 30% fibroglandular content by volume). It is difficult to determine the true average dielectric properties in heterogeneous breast phantoms. Hence, the accuracy, $\Delta \varepsilon_{r}$, is not shown because the accuracy, $\Delta \varepsilon_{r}$, is of limited value when the true average dielectric properties are not known exactly.

In the heterogeneous breast phantom (rows 1–5) with 10% glandular structures by volume, the Gaussian Energy, $\phi_{\mathrm{GSS}}^{G}$, and the Central Moment, $\phi_{\mathrm{ACM}}^{S}$, perform very similarly, selecting an average dielectric properties value of within $\Delta \varepsilon_{r}=0.25$ for all five tumour models. The SCR for the chosen images for all metrics in this case are within 0.3 dB of each other. The Modified Laplacian, $\phi_{M}^{L}$, selects almost the same images except for the fourth tumour model where the wrong image is selected. As can be seen in Table 3, the Modified Laplacian, $\phi_{M}^{L}$, selects an image with a higher SMR and slightly lower SCR than the Gaussian Energy, $\phi_{\mathrm{GSS}}^{G}$, and the Central Moment, $\phi_{\mathrm{ACM}}^{S}$.

**Figure 5:**
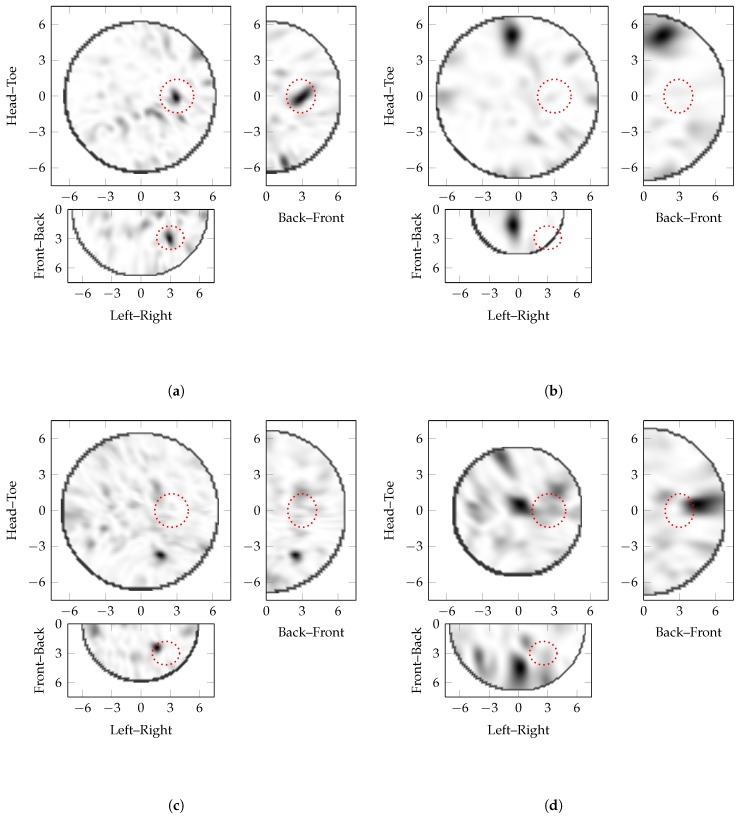
Shown are coronal, sagittal and transverse slices of images of the tumour model $T_{4}$. (**a**,**b**) are in a phantom with 10% glandular structures by volume and (**c**,**d**) are in a phantom with 30% glandular structures by volume. (**a**,**c**) are the images selected by the Gaussian Energy, $\phi_{\mathrm{GSS}}^{G}$; and (**b**,**d**) are the images selected by the Modified Laplacian, $\phi_{M}^{L}$. The actual target location is marked by the dotted, red ellipse in each slice.

Figure 5a,b show the images selected by the Gaussian Energy, $\phi_{\mathrm{GSS}}^{G}$, and the Modified Laplacian, $\phi_{M}^{L}$, respectively. Although the Gaussian Energy, $\phi_{\mathrm{GSS}}^{G}$, weights an image where the tumour target is clearly identifiable in the correct location, the Modified Laplacian, $\phi_{M}^{L}$, weights an alternative image more highly. The image shown in Figure 5b is reconstructed with lower average dielectric properties and exhibits the characteristics identified earlier, where the response in the image is much closer to the skin.

As the volume fraction of glandular tissue increases to 20% and 30%, the quality of the optimal image decreases due to increased reflections from other structures within the breast. In particular, the maximum response within the image is much further from the true tumour model location and the tumour model location is not correctly determined. The Gaussian Energy, $\phi_{\mathrm{GSS}}^{G}$, and the Central Moment, $\phi_{\mathrm{ACM}}^{S}$, again reward similar images for all tumour models.

Coronal, sagittal and transverse slices of the reconstructed images of $T_{4}$ in a phantom with 30% glandular structures by volume are shown in Figure 5c,d, corresponding to the image most rewarded using the Gaussian Energy, $\phi_{\mathrm{GSS}}^{G}$, and the Modified Laplacian, $\phi_{M}^{L}$, respectively. Although, in this very heterogeneous breast phantom, no image is reconstructed that accurately identifies the tumour target location, the Modified Laplacian, $\phi_{M}^{L}$, rewards an image reconstructed with lower average dielectric properties with a large apparent response close to the skin. Both the Gaussian Energy, $\phi_{\mathrm{GSS}}^{G}$, and the Modified Laplacian, $\phi_{M}^{L}$, reward an image with a bright response in this case, although the location of this response is approximately 40 mm away from the true tumour target location.

Many FQMs are found to have very similar performance in this work, in homogeneous and heterogeneous breast phantoms. This is expected as most FQMs are designed for the same purpose and it is indicated that FQMs are appropriate fitness functions for estimating average dielectric properties.

For example, gradient-based metrics, $\phi^{G}$:

- can be calculated easily from the image using simple and well-known kernels in two and three dimensions;
- have a well-understood method of action as differentiation is analogous to high-pass filtering;
- and identify the optimal image in heterogeneous phantoms with different tumour sizes.

Additionally, the Gaussian Energy, $\phi_{\mathrm{GSS}}^{G}$, is shown in this work to be a suitable cost function in three-dimensional images with realistic artefact removal.

## 6. Conclusions and Future Work

Microwave breast imaging assumes that an estimate of the effective average dielectric properties can be found and used to synthetically focus the backscattered signals. Errors in the estimate of effective average dielectric properties reduce coherent addition at dielectric scatterer locations, leading to poorer image quality and increased localisation errors. Patient studies with microwave radar imaging to date have used a single estimate determined in advance from experimental studies or published dielectric properties studies.

Due to the importance of the effective average dielectric properties on image quality, parameter search algorithms have been proposed as a method to determine this patient-specific value. Parameter search algorithms rely on correctly reconstructed images having identifiable properties. These methods do not rely on a given propagation path being representative of the imaging volume as a whole, and are not affected by problems with multipath propagation unlike the previously used transmission-based methods.

This work identifies properties of images reconstructed with over- and underestimated average dielectric properties. In particular, when underestimating the effective average dielectric properties, it was found that the apparent location of the response moves towards the edge of the imaging domain and that the spatial frequency content of the microwave breast image decreases. Conversely, as the effective average dielectric properties were overestimated, the apparent location of the response moves towards the centre of the imaging domain and the spatial frequency content of the microwave breast image increases. FQMs, common in digital camera autofocus, were identified as potentially suitable fitness functions, as they estimate the spatial frequency content of images.

Many FQMs were found to reward high quality images, indicating that this type of metric is suitable for average dielectric properties estimation. In particular, gradient-based metrics are computationally simple, have a well-understood method of action and are suitable fitness functions, such as the Gaussian Energy, $\phi_{\mathrm{GSS}}^{G}$. These results from dielectrically heterogeneous phantoms with different tumour sizes indicate that parameter search algorithms using FQMs as fitness functions are suitable for estimating the average dielectric properties for microwave breast imaging.

Future work should evaluate clinical data gathered from patient studies, both healthy and with disease, to ensure that the algorithms are robust to a wide range of tissues and clinical scenarios, including healthy cases.

## Figures and Tables

**Table 1:** Summary of the names, abbreviations and methods of action. $\mathrm{var}\left[X\right]$ represents the variance of *X* across the imaging area, and $\langle X\rangle$ represents the mean of *X* across the imaging area.

Name	Equation
AC–DC Ratio [71]	$\phi_{R}^{F}=\langle \frac{\sum_{\left(n,m)\neq (0,0\right)}F_{x,y}{\left(n,m\right)}^{2}}{F_{x,y}{\left(0,0\right)}^{2}}\rangle$
AC–DC Reduced Ratio [72]	$\phi_{\mathrm{RR}}^{F}=\langle \frac{\sum_{\left(n,m\right)\in P_{r}}F_{x,y}{\left(n,m\right)}^{2}}{F_{x,y}{\left(0,0\right)}^{2}}\rangle$
Absolute Gradient [73]	$\phi_{\mathrm{DMA}}^{G}=\langle \max_{D\in \{X,Y\}}\left|I_{D}^{D}\left(x,y\right)\right|\rangle$
Squared Gradient [73]	$\phi_{\mathrm{DMS}}^{G}=\langle \max_{D\in \{X,Y\}}{\left|I_{D}^{D}\left(x,y\right)\right|}^{2}\rangle$
Brenner Gradient [60,73,74,75]	$\phi_{\mathrm{BMS}}^{G}=\langle \max_{D\in \{X,Y\}}{\left|I_{D}^{B}\left(x,y\right)\right|}^{2}\rangle$
Gradient Energy [66,76,77]	$\phi_{\mathrm{DSS}}^{G}=\langle I_{X}^{D}{\left(x,y\right)}^{2}+I_{Y}^{D}{\left(x,y\right)}^{2}\rangle$
Gaussian Energy [78,79]	$\phi_{\mathrm{GSS}}^{G}=\langle I_{X}^{G}{\left(x,y\right)}^{2}+I_{Y}^{G}\left(x,y\right)\rangle$
Tenengrad Mean [69,74,77,80,81]	$\phi_{\mathrm{TM}}^{G}=\langle \max_{D\in \{X,Y\}}I_{D}^{T}{\left(x,y\right)}^{2}\rangle$
Tenengrad Variance [80]	$\phi_{\mathrm{TV}}^{G}=\mathrm{var}\left[\max_{D\in \{X,Y\}}I_{D}^{T}\right]$
Laplacian Energy [63,69,70,82]	$\phi_{E}^{L}=\langle \left|L*I(x,y)\right|\rangle$
Modified Laplacian [83]	$\phi_{M}^{L}=\langle \left|L_{x}*I\left(x,y\right)\right|+\left|L_{y}*I\left(x,y\right)\right|\rangle$
Diagonal Laplacian [84]	$\phi_{D}^{L}=\langle \phi_{M}^{L}\left(x,y\right)+\;\;\;\;\sum_{n\in \{1,2\}}\;\;\left|L_{dn}*I\left(x,y\right)\right|\rangle$
Laplacian Variance [80]	$\phi_{V}^{L}=\mathrm{var}\left[L*I\right]$
Variance [65,66,67,68,73,74,75,77,81,85]	$\phi_{V}^{S}=\mathrm{var}\left[I(x,y)\right]$
Normalised Variance [72,73,74]	$\phi_{\mathrm{VN}}^{S}=\frac{1}{\langle I(x,y)\rangle }\mathrm{var}\left[I(x,y)\right]$
Localised Variance [80]	$\phi_{\mathrm{VL}}^{S}=\mathrm{var}\left[L_{v}\left(x,y\right)\right]$
Contrast [86]	$\phi_{C}^{S}=\langle \sum_{i\in W}\sum_{j\in W}I\left(x,y\right)\;-\;I\left(x+i,y+j\right)\;\rangle$
Mean Ratio [81]	$\phi_{R}^{S}=\langle \max\left\{\frac{\mu (x,y)}{I(x,y)},\frac{I(x,y)}{\mu (x,y)}\right\}\rangle$
Entropy [65,73,74,75,85,87]	$\phi_{\mathrm{HE}}^{S}=H\left(I_{H}\right)$
Central Moment [41,88]	$\phi_{\mathrm{ACM}}^{S}=\sum_{k}\left|k-\langle I\rangle \right|P_{k}$
Absolute Detail Sum [41,87,89]	$\phi_{\mathrm{AS}}^{W}=\langle \sum_{n\in \{\mathrm{LH},\mathrm{HL},\mathrm{HH}\}}\left|W_{n}^{1}\left(x,y\right)\right|\rangle$
Detail Variance [87,89]	$\phi_{V}^{W}=\langle \sum_{n\in \{\mathrm{LH},\mathrm{HL},\mathrm{HH}\}}\mathrm{var}\left[W_{n}^{1}\right]\rangle$
Detail–Coarse Ratio [41,87]	$\phi_{R}^{W}=\langle \frac{W_{\mathrm{LH}}^{1}{\left(x,y\right)}^{2}+W_{\mathrm{HL}}^{1}{\left(x,y\right)}^{2}+W_{\mathrm{HH}}^{1}{\left(x,y\right)}^{2}}{W_{\mathrm{LL}}^{1}{\left(x,y\right)}^{2}+W_{\mathrm{LL}}^{2}{\left(x,y\right)}^{2}+W_{\mathrm{LL}}^{3}{\left(x,y\right)}^{2}}\rangle$

**Table 2:** Evaluation of performance of all metrics in homogeneous scenarios. Ranks are shown in parentheses, within each method of action for each individual criterion. Two overall ranks are shown, first within each method of action and then for all metrics (local/global). The top performing metrics are shown in **bold**.

Metric	$\Delta \varepsilon_{r}$	Δr	SMR$\left(I_{\varepsilon_{r}^{B^{\prime }}}\right)$	SCR$\left(I_{\varepsilon_{r}^{B^{\prime }}}\right)$	Ranks
$\phi_{R}^{F}$	2.5 (1)	10.9 (1)	7.6 (1)	3.8 (1)	(**1/21)**
$\phi_{\mathrm{RR}}^{F}$	3.0 (2)	13.3 (2)	5.0 (2)	2.5 (2)	(2/22)
$\phi_{\mathrm{GSS}}^{G}$	1.4 (2)	4.6 (1)	18.3 (1)	8.5 (1)	(**1/2)**
$\phi_{\mathrm{DMA}}^{G}$	1.3 (1)	6.6 (2)	16.7 (2)	8.0 (2)	(2/7)
$\phi_{\mathrm{TM}}^{G}$	1.4 (3)	7.4 (3)	14.5 (3)	7.1 (3)	(3/8)
$\phi_{\mathrm{DMS}}^{G}$	1.5 (4)	7.4 (4)	14.5 (4)	7.1 (4)	(4/10)
$\phi_{\mathrm{DSS}}^{G}$	1.5 (4)	7.4 (4)	14.5 (4)	7.1 (4)	(4/10)
$\phi_{\mathrm{BMS}}^{G}$	1.5 (6)	7.6 (6)	14.5 (6)	7.1 (6)	(6/15)
$\phi_{\mathrm{TV}}^{G}$	1.9 (7)	8.9 (7)	11.8 (7)	5.7 (7)	(7/18)
$\phi_{M}^{L}$	1.5 (1)	7.3 (1)	14.5 (1)	7.0 (4)	(**1/9)**
$\phi_{D}^{L}$	1.5 (3)	7.3 (2)	14.5 (2)	7.0 (3)	(2/12)
$\phi_{E}^{L}$	1.5 (3)	7.6 (4)	14.4 (4)	7.0 (2)	(4/16)
$\phi_{V}^{L}$	1.5 (3)	7.6 (4)	14.4 (4)	7.0 (2)	(4/16)
$\phi_{\mathrm{ACM}}^{S}$	0.7 (1)	5.2 (1)	17.1 (2)	8.3 (1)	(**1/1)**
$\phi_{V}^{S}$	0.8 (2)	5.5 (3)	17.1 (3)	8.3 (2)	(2/**3)**
$\phi_{\mathrm{VL}}^{S}$	1.3 (5)	5.9 (4)	17.5 (1)	8.2 (3)	(3/**4)**
$\phi_{\mathrm{VN}}^{S}$	1.3 (4)	5.2 (2)	16.4 (5)	7.9 (5)	(4/6)
$\phi_{C}^{S}$	1.3 (3)	6.6 (5)	16.7 (4)	8.1 (4)	(4/**5)**
$\phi_{R}^{S}$	2.3 (6)	9.8 (6)	7.5 (7)	3.6 (7)	(6/20)
$\phi_{\mathrm{HE}}^{S}$	2.4 (7)	11.0 (7)	7.8 (6)	3.9 (6)	(6/19)
$\phi_{V}^{W}$	1.5 (2)	7.3 (1)	14.5 (1)	7.0 (1)	(**1/12)**
$\phi_{\mathrm{AS}}^{W}$	1.5 (2)	7.3 (2)	14.5 (2)	7.0 (2)	(2/14)
$\phi_{R}^{W}$	5.0 (3)	31.0 (3)	0.0 (3)	0.0 (3)	(3/23)

**Table 3:** Δr, $\mathrm{SMR}(I_{\varepsilon_{r}^{B^{\prime }}})$ and $\mathrm{SCR}(I_{\varepsilon_{r}^{B^{\prime }}})$ evaluated for spherical targets of increasing diameter in phantoms of increasing heterogeneity.

	Δr (mm)			SMR$\left(I_{\varepsilon_{r}^{B^{\prime }}}\right)$ (dB)			SCR$\left(I_{\varepsilon_{r}^{B^{\prime }}}\right)$ (dB)		
	$\phi_{\mathrm{GSS}}^{G}$	$\phi_{M}^{L}$	$\phi_{\mathrm{ACM}}^{S}$	$\phi_{\mathrm{GSS}}^{G}$	$\phi_{M}^{L}$	$\phi_{\mathrm{ACM}}^{S}$	$\phi_{\mathrm{GSS}}^{G}$	$\phi_{M}^{L}$	$\phi_{\mathrm{ACM}}^{S}$
**10% het.**									
d = 5.3	12	11	12	1.9	1.7	1.9	15.6	15.7	15.6
d = 7.8	14	14	14	3.9	3.6	3.9	17.8	17.8	17.8
d = 10.9	1	1	1	1.5	1.9	1.9	16.6	16.6	16.6
d = 13.1	2	63	2	0.5	2.9	0.5	14.3	14	14.3
d = 20.2	8	8	8	2.3	1.9	2.3	13.7	13.7	13.7
**20% het.**									
d = 5.3	83	83	83	3.5	3.5	3.5	14.9	14.9	14.9
d = 7.8	21	76	21	1.1	0.7	1.1	14.6	14	14.6
d = 10.9	45	52	45	0.9	0.8	0.9	12.8	12.8	12.8
d = 13.1	3	4	3	3.8	3.6	3.8	17.9	17.9	17.9
d = 20.2	7	8	7	2.4	2.5	2.4	15.5	15.6	15.5
**30% het.**									
d = 5.3	36	16	36	0.1	0.6	0.1	11.8	10.4	11.8
d = 7.8	21	21	21	0.2	0.2	0.2	12.1	12.1	12.1
d = 10.9	29	29	29	0.5	0.9	0.5	11.9	12.1	11.9
d = 13.1	39	28	39	0.6	1	0.6	13	11.1	13
d = 20.2	13	9	13	1.2	1.3	1.2	16.5	15.6	16.5
